# Potential Drug-drug Interactions in Post-CCU of a Teaching Hospital

**Published:** 2013

**Authors:** Mohammad Haji Aghajani, Mohammad Sistanizad, Mohammad Abbasinazari, Mahdieh Abiar Ghamsari, Ladan Ayazkhoo, Olia Safi, Katayoon Kazemi, Mehran Kouchek

**Affiliations:** a *Department of Cardiology, Imam Husain Educational Hospital, Shahid Beheshti University of Medical Sciences, Tehran, Iran.*; b *Department of Clinical Pharmacy, Faculty of Pharmacy, Shahid Beheshti University of Medical Sciences, Tehran, Iran. *; c *Imam Husain Educational Hospital, Shahid Beheshti University of Medical Sciences, Tehran, Iran. *; d *Anesthesiology Research Center, Shahid Beheshti University of Medical Sciences, Tehran, Iran.*; e *Pharmaceutical Sciences Research Center, Shahid Beheshti University of Medical Sciences, Tehran, Iran. *

**Keywords:** Post-CCU, Drug interaction, Patient safety, Adverse drug effect

## Abstract

Drug-drug interactions (DDIs) can lead to increased toxicity or reduction in therapeutic efficacy. This study was designed to assess the incidence of potential drug interactions (PDI) and rank their clinical value in post coronary care unit (Post-CCU) of a teaching hospital in Tehran, Iran.

In this prospective study, three pharmacists with supervision of a clinical pharmacist actively gathered necessary information for detection of DDIs. Data were tabulated according to the combinations of drugs in treatment chart. Verification of potential drug interactions was carried out using the online Lexi-Interact™ 2011.

A total of 203 patients (113 males and 90 females) were enrolled in the study. The mean age of patients was 61 ± 12.55 years (range = 26-93). A total of 90 drugs were prescribed to 203 patients and most prescribed drugs were atorvastatin, clopidogrel and metoprolol. Mean of drugs was 11.22 per patient. A total of 3166 potential drug interactions have been identified by Lexi- Interact™, 149 (4.71%) and 55 (1.73%) of which were categorized as D and X, respectively. The most serious interactions were clopidogrel+omeprazole and metoprolol+salbutamol.

Drug interactions leading to serious adverse effects are to be cautiously watched for when multiple drugs are used simultaneously. In settings with multiple drug use attendance of a pharmacist or clinical pharmacist, taking the responsibility for monitoring drug interactions and notifying the physician about potential problems could decrease the harm in patient and increase the patient safety.

## Introduction

An interaction occurs when the effects of one drug are changed by the presence of another drug, herbal medicine, food, drink or by some environmental chemical agents (1). Drug-drug interaction (DDI) is a type of adverse drug effect (ADE) that is important to be recognized and prevented due to its consequences. There is a difference between a drug side effect and a drug interaction. Drug side effect is related to a single drug, but an interaction is due to two or more drugs administration. DDIs are big problems and they arise in numerous ways (2, 3).

The risk of occurrence and severity of a DDI depends on several factors, including the number of drugs prescribed, duration of treatment, patient age and stages of disease. Patients that require a large number of drugs, long time of treatment, with physiological aging changes or certain diseases such as renal failure, shock(4, 5), hepatic diseases such as cirrhosis or acute viral hepatitis, are considered as high risk for severe drug interactions (6-9).

In assessment of these PDIs, especially those with clinical value, the severity of the effect and the level of evidence must be considered. Potential for drug interaction is higher with cardiac drugs (10) and there are no studies reporting actual incidence of DDIs in cardiology center in the Iranian setting. Hence, this study was designed to assess the incidence of PDIs and ranking their clinical value based on medical prescriptions in post coronary care unit (Post-CCU) of a teaching hospital in Tehran, Iran.

## Experimental

*Study design and setting*

This cross-sectional study was conducted in the post-CCU of Imam Husain multispecialty teaching hospital affiliated to Shahid Beheshti Medical University, Tehran, Iran from April 2011 to September 2011. Three pharmacists with supervision of a clinical pharmacist actively gathered necessary information for detection of DDIs.

*Patients*

All patients admitted to the post CCU were screened for eligibility to enter the study. Patients with more than a 48 h stay were included in the study. Informed consent was not considered necessary by the ethics committee of involved institutions.

*Data collection*

Data were collected from inpatient case notes, treatment charts, laboratory data reports and patient interview. All the necessary and relevant information including age, gender, cause of admission, drug name, dosage form, dosage, route of administration and timing of the administration was collected for each patient in designed questionnaires.

*Data evaluation and analysis*

Data were tabulated according to the combinations of drugs in treatment chart. Intravenous fluids, nutritional supplements, insulin and vitamins were excluded from this assay. Verification of potential drug interactions was carried out using the software Lexi-Interact™ 2011 (11). This verification procedure took place at the end of the study and researchers were not aware of the potential drug interactions during data collection. The study did not envisage methods to investigate the actual occurrence of interactions.

The assessment of PDI risk was made by evaluating the severity of the effect supplied via the Lexi-interact™ 2011. The occurrence and severity of potential drug interactions were evaluated by cross-checking each patient’s prescription profile by Lexi-Interact™ drug interaction checking database. According to the Lexi-interact™, the risk was ranked into A, B, C, D and X (Table 1). The progression from A to X is accompanied by increased urgency for responding the data. In general, A and B monographs are of academic, but not clinical concern. Monographs rated C, D, or X always require the user’s attention (11).

**Table 1:** Classification of importance of interactions.

Risk Rating	Action	Description
A	***No Known Interaction***	**Data have not demonstrated either pharmacodynamic or pharmacokinetic interactions**
B	***No Action Needed***	**May interact with each other, but there is no evidence of clinical concern**
C	***Monitor Therapy***	**The benefits of concomitant use of these two medications usually outweigh the risks**
D	***Therapy Modification***	**Assess whether the benefits of concomitant therapy outweigh the risks or not**
X	***Avoid Combination***	**The risks associated with concomitant use outweigh the benefits**

## Results

**Figure 1:**
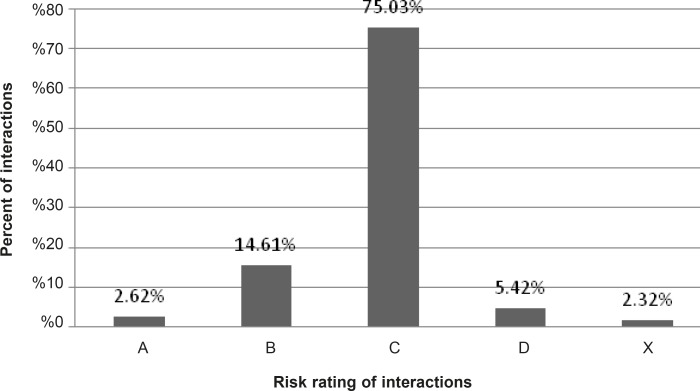
Percent of potential drug-drug interactions in post-CCU ward of a teaching hospital (total 3360 PDIs).

A total of 203 patients (113 males and 90 females) were enrolled in the study during the study period. The mean age of patients was 61 ± 12.55 years (range = 26-93). Ninety drugs were used to 203 patients and most prescribed drugs were atorvastatin, clopidogrel and metoprolol. Mean of drugs per treatment chart was 11.22 ± 3.91. In total, 3360 potential drug interactions (16.5 PDI per treatment chart) have been identified by Lexi-Interact™, which 5.42% and 2.32% were categorized as D and X, respectively (Figure 1). The most common potential drug interactions are shown in Table 2.

**Table 2:** The most common potential drug interactions based on risk of interaction in post-CCU ward.

**Risk Rating ofInteraction**	**Drug pairs**		**Observed No.**	**Percent in3360 PDI **	**Level ofEvidence **
**A**	Aspirin	spironolactone	37	1.1%	Good
	Clopidogrel	magnesium hydroxide	22	0.65%	Good
**B**	atorvastatin	clopidogrel	112	3.33%	Good
	Aspirin	nitroglycerine	114	3.4%	Good
**C**	Aspirin	clopidogrel	128	3.81%	Good
	atorvastatin	omeprazole	73	2.17%	Poor
**D**	Aspirin	warfarin	15	0.44%	Excellent
	pantoprazole	clopidogrel	16	0.47%	Fair
**X**	Clopidogrel	omeprazole	71	2.11%	Fair
	Carvedilol	salbutamol	5	0.15%	Fair

## Discussion

The results of this study showed 78 contraindicated and 182 risk D combinations in 203 medication profiles which means 1.28 category D or X interaction per profile. Studies have shown that the increase in both length of hospital stay and cost of hospitalization could be related to possible adverse events resulting from drug interactions. For instance, a drug-related problem may demand extra lab tests or a symptomatic treatment that could lead to a prolongation of hospital stay and increased cost (12, 13).

DDIs are more likely to occur in hospital settings, where multiple drugs are often prescribed concomitantly. When various drugs are being administered, there is a probability of DDIs as one drug can increase or decrease the effect of another drug or other serious reactions, resulting in increased toxicity or reduced therapeutic efficacy (13, 14). An Australian study found that about 10% of hospital admissions were drug-related, of which 4.4% were due to drug interactions (15). In a review of drug related admissions in Australia, 6-7% of emergency admissions, 12% of all admissions to medical wards and 15-22% of all emergency admissions among the elderly were drug related. Between 32% to 69% of drug-related admissions were reported as definitely or possibly preventable (16). In another study in the US, a total of 226 potential drug interactions (PDIs) were found in 89 patients (47%), with 50% of DDIs being related to emergency department treatment. The risk of a DDI rose from 13% for patients taking 2 medications to 82% for patients taking 7 or more medications (17).

In another study in 2005, Nazari *et al. *evaluated the drug interactions in 567 ICU prescriptions and found 413 DDIs. There was a direct relationship between the number of drugs per prescription and the frequency of interactions (18). We found that Drug-related problems including DDIs are significant and expensive public health problems (6, 16, 19). About 32-69% of these problems were considered possibly or probably preventable (16). As drug prescription is the most common remedial act in treatment of patients, recognition and prevention of possible DDIs are of great value in drug administration or drug development. Lack of knowledge in this field can cause displeasure with care and reduced quality of life and also accounts for regular visits to the emergency departments.

Patients with cardiovascular diseases are particularly vulnerable to DDIs due to their advanced age, polypharmacy and the influence of heart disease on drug metabolism. The DDI potential for a particular cardiovascular drug varies with the individual, the disease being treated and the extent of exposure to other drugs (20).

Ortele *et al.*, studied drug-drug interactions in a Swiss primary and secondary acute care hospital and showed that on average, each patient encountered 5 DDIs, 5% of which were classified in category 1 (contraindicated), 3% in category 2, 53% in category 3, 8% in category 4 and 31% in category 5 (21).

Drug interactions leading to serious adverse effects are to be cautiously watched for when multiple drugs are used simultaneously (22). It is important for the physician to be aware of these interactions. Although in many instances the adverse interaction does not reach a magnitude of recognizable clinical expression, rarely it can result in a serious adverse outcome.

Adverse drug interactions may lead to increased toxicity, decreased efficacy or both. Most of these interactions could be managed by monitoring possible adverse effects or simply by changing one of medications in contraindicated combination. For example, the inhibition of platelet aggregation by clopidogrel is entirely due to an active metabolite. Clopidogrel is metabolized to this active metabolite in part by CYP2C19. In patients receiving clopidogrel and omeprazole concomitantly, omeprazole decreases the effects of clopidogrel by affecting hepatic enzyme CYP2C19 metabolism. This interaction could be managed by changing omeprazole to another proton pump inhibitor with lower inhibitory effect on hepatic enzymes or using H2 blockers like ranitidine (11, 23).

The possibility of interaction with non-prescription drugs, herbal or alternative medicines or food should be also borne in mind. Increased risk of drug-induced toxicity or therapeutic failure can occur when a new drug is added to a treatment regimen. It is impossible to remember all possible drug interactions. A ready to refer checklist or using an interaction checking software is useful as a handy reference.

In some institutions, computerized drug-drug interaction surveillance systems have been implemented. These systems yield a large number of false-positive alerts. Clinically insignificant alerts can lead to alert fatigue. For example, a clinician receives many insignificant alerts and then does not take preventive action when a clinically significant alert occurs due to the oversight from alert volume (24, 25). In addition, the interpretation of drug-drug interaction alerts, without clear clinical relevance of the interaction, may lead to differences in the perception of the interaction’s seriousness and lack of necessary interventions (26). Research has shown that a small number of alerts require intervention, and insignificant alerts should be suppressed to prevent alert fatigue (27, 28).

As pharmacists are aware of the side effects of medications and mechanisms of drug interactions and have knowledge and ability to relate unexpected symptoms experienced by patients to possible adverse effects of their drug therapy, attendance of pharmacist in the treatment team of patients could be helpful (28). Especially, in settings with multiple drug use like ICU and CCU attendance of pharmacist or clinical pharmacist, appropriately applying molecular mechanisms by which drugs interact to specific patient and taking responsibility for monitoring drug interactions and notifying the physician about potential problems, could decrease harm in patient and increase the patient’s safety.
